# The effects of intensive trapping on invasive round goby densities

**DOI:** 10.1371/journal.pone.0301456

**Published:** 2024-05-08

**Authors:** Maya S. Enriquez, Lily M. Hall, Noland O. Michels, Emily R. Fleissner, Allen F. Mensinger

**Affiliations:** Department of Biology, University of Minnesota-Duluth, Duluth, Minnesota, United States of America; MARE – Marine and Environmental Sciences Centre, PORTUGAL

## Abstract

The round goby (*Neogobius melanostomus*) is an invasive benthic fish first introduced to the Laurentian Great Lakes in 1990 that has negatively impacted native fishes through increased competition for food and habitat, aggressive interactions, and egg predation. While complete eradication of the round goby is currently not possible, intensive trapping in designated areas during spawning seasons could potentially protect critical native fish spawning habitats. Baited minnow traps were spaced 10 meters apart in shallow water along a 100-meter stretch of shoreline within the Duluth-Superior Harbor during the round goby breeding period (June to October) with captured round gobies removed from interior traps (N = 10) every 48 hours. These traps were bracketed by two pairs of reference traps deployed weekly for 48 hours, from which round gobies were also tagged and released. The number of round gobies captured in the interior traps declined by 67% compared to reference traps over the course of the study, with extended periods of no captures. The tagged round gobies showed high site affinity, with 82.8% of tagged fish recaptured at the previous release site. The results indicate that even at open water sites, which allow natural migration of round gobies into the area, extensive trapping could reduce local population numbers.

## Introduction

The Laurentian Great Lakes have been designated an aquatic invasive species hotspot due to a wide range of exotic organisms that recently have been introduced and threaten native wildlife [1]. The round goby (*Neogobius melanostomus)* is a benthic fish native to the Ponto-Caspian Sea that was transported to the Laurentian Great Lakes in ballast water discharged from transoceanic shipping vessels [2, 3]. Since their initial discovery in the St. Clair River [4], round gobies have established populations in all the Laurentian Great Lakes and continue to spread into surrounding tributaries [2, 4–6].

The successful expansion of the round goby has been attributed to several factors, including their physiology, fecundity, and behavior. Round gobies can withstand large fluctuations in salinity, permitting survival in oceanic ballast water which could allow for further spread into brackish water estuaries along the North American coast [7]. Round gobies also reproduce multiple times each year, giving them an advantage over many Great Lakes native fishes that spawn yearly [2, 4, 8, 9], and outcompete native fishes for space and food [9–12]. Additionally, round gobies negatively impact native fish populations by feeding on the eggs of lake sturgeon (*Acipenser fulvescens*) [13], mottled sculpin (*Cottus bairdii*) [4], lake trout (*Salvelinus namaycush*) [14, 15] and smallmouth bass (*Micropterus dolomieu*) [16]. Although they are one of the few organisms that prey on the invasive zebra dreissenid mussel (*Dreissena polymorpha*) [17], this benefit is offset by potential biomagnification of polychlorinated biphenyls (PCBs) from mussel ingestion, which could lead to increased toxins in game fishes [18–20].

Complete eradication of the round goby is currently not possible due to their abundance and widespread population in the Laurentian Great Lakes and lack of suitable control measures. It is difficult to eliminate invasive species once they are established and therefore, most efforts are focused on curtailing invasion into new ecosystems. Shipping regulations have been established to reduce the presence of species in ballast water by exchanging freshwater for high salinity oceanic water before entry to the Laurentian Great Lakes [21], as well as mandates that enforce water ballast management plans and support on-board removal of invasive species through physical (i.e. filtration, UV, heat) or chemical removal [22]. Migration barriers such as carbon dioxide infusion at the critical bottlenecks including lock navigation channels on rivers [23] and electric gated barriers [24] also are being examined.

Removal methods such as trawling or seining are complicated by the round goby’s preference for rocky habitat. Trawling may be useful in river and canal habitats with soft sediment, but intermittent wood, rocks, or other detritus can damage net bottoms and is labor and equipment intensive [5, 25]. Additionally, targeted removal with SCUBA is cost prohibitive [26]. Electrofishing is normally ineffective, as the fish will not float to the surface. However, it is considered more effective than angling or minnow traps in a boulder dominated bank [27]. Bioacoustic traps have been suggested for soniferous fish, such as the round goby [28, 29] but have not been developed further than proof-of-concept [30]. Alternatively, targeted trapping may be a viable method for temporarily reducing round goby densities in areas of high interest and during critical time periods, such as native fish breeding seasons, and may be more cost effective than seining, electrofishing, or SCUBA collecting [26, 31].

Additionally, round goby movement may make the fish more susceptible to trapping as fish move from deeper water to the shallows in the spring [32–34], with larger gobies colonizing shallow areas and smaller gobies pushed to less preferable deeper water [33, 35, 36]. Round gobies have high site affinity, with previous studies finding an average home range of 5 m^2^ [35] and mark recapture studies showing a high percentage of recaptures in the Duluth Superior Harbor with limited along-shore migrations rarely exceeding 25 m from initial capture sites [33]. Off shore migrations may be of greater distances [37], however the seasonal migration pattern may allow for targeted removal during peak migration times. Additionally, it may be possible by removing large on-site breeding adults and placing traps in strategic areas to capture on or along-shore migrants, pressure could be lessened on native fishes in an area.

The purpose of this study was to quantify the efficacy of intensive trapping on the local round goby population along one 100 m shoreline of shallow, rocky shoreline within the Duluth Superior Harbor. This area has a high density of large, sexually mature gobies with few native fishes [33]. We hypothesized that regular trapping every 48 hours over the course of the summer season would reduce the overall number and size of gobies in the area. We examined if repeated removal reduces the relative abundance in a localized area, if these removals altered fish size and gender ratio and quantified the degree of along-shore movement by gobies near the capture locations.

## Materials and methods

### Study site

**Fig 1:**
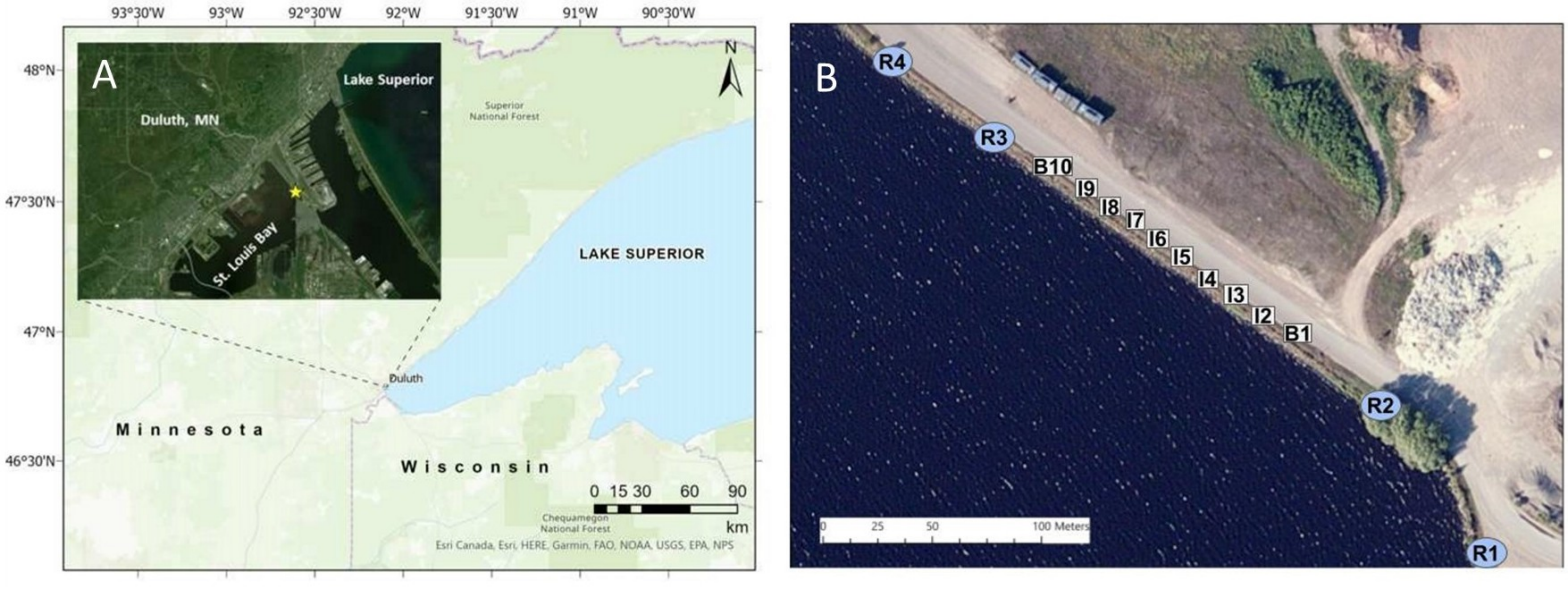
A) The Duluth-Superior Harbor is located at the westernmost part of Lake Superior. The study site is indicated by the star. B) The 100 m study site in the Duluth-Superior Harbor at Rice’s Point, 46°45’14.0"N 92°06’28.8"W. Reference traps (blue circles R1 through R4) were placed 50 m and 20 m to the left and right of the interior traps (numbered white boxes) which were spaced 10 m apart. Experimental traps include border traps B1 and B10 and internal traps I2 through I9.. Both figures were obtained from Esri, USGS and subsequently modified.

The Duluth-Superior Harbor is located at the mouth of the Saint Louis River, at which point the 309 km long river empties into a 4,856-hectare freshwater estuary prior to water flowing into Lake Superior. The study site was based at Rice’s Point, 46°45’14.0" N 92°06’28.8" W (Fig 1), which is in the harbor and is the site of two heavy industrial plants. The shoreline and shallows in this area are characterized by an array of interstitial spaces created by a man-made backfill of rock, metal, and concrete over a sandy bottom and was relatively uniform throughout the study area. Water depths are relatively shallow (<1 m) before increasing to approximately 8 m at the dredged navigational channel 50 m offshore [NOAA Chart 14975, 2020]. The site contains a high density of round gobies with very few native benthic species [33].

### Sampling methods

Fourteen sampling stations (Fig 1B) were established in shallow water along 200 m of shoreline at Rice’s Point between June 6th to October 29th, 2019. Galvanized mesh minnow traps (n = 14, 44 cm long with circular ends 18.5 cm in diameter each containing a 2.5 cm diameter opening) were placed at depths ranging from 0.5 to 1.5 m with fixed anchor points within 5 m of the shoreline. Traps were baited with thawed fish scraps consisting primarily of salmon and trout species (Northern Waters Smokehaus, Duluth, MN, USA). Interior traps (n = 10) were placed 10 m apart in the center of the experimental area and were designated as border (traps B1 and B10) or internal (traps I2 through I9) traps. A temperature and light data logger (HOBO Pendant^®^ Temperature /Light Data Logger; Onset Computer Corporation, Bourne, MA, USA) was placed on an interior trap (I5) to record daily water temperature. Reference traps (n = 4) were placed along the shore 20 m and 50 m northwest or southeast of experimental Traps B1 and B10. Interior traps were checked every 48 hours, the contents collected, re-baited and then deployed. Round gobies that were captured in interior traps were transported to lab facilities or euthanized via overdose of MS-222 (Western Chemical Inc., Ferndale, WA, United States) while other species were released at the same location. Reference traps were baited and deployed every 6 or 8 days and removed after 48 hours. Round gobies caught in reference traps were released at the same location with 167 fish tagged with Visible Implant Alpha Tags (Northwest Marine Technology, Shaw Island, WA, USA) from June 21st to October 1st, 2019. These tags were shown to have high retention rates in the round goby with numbers visible for at least 15months [33]. Round goby total length (TL) and sex was determined. All experiments conformed with University of Minnesota IACUC protocols (1801-35507A) and trapping was permitted under Minnesota Department of Natural Resources invasive species permit #491.

### Analysis

Catch Per Unit Effort (CPUE) was defined as the number of round gobies captured in one trap over a 48-hour period. The data were not normally distributed (Shapiro-Wilk test, (P < 0.050) and nonparametric tests were used for analysis. The four reference traps, two boarder traps and 8 internal taps were intracompared within each category. The proportion of males from each trap category was quantified monthly throughout the study. The along shore movement of tagged gobies from the reference traps were quantified throughout the study area. CPUE and total length of round gobies in interior, border, and reference traps were compared with a Kruskal-Wallis ANOVA and with Holm-Sidak post hoc tests. All statistical analyses were performed using SigmaPlot software (Version 14).

## Results

**Fig 2:**
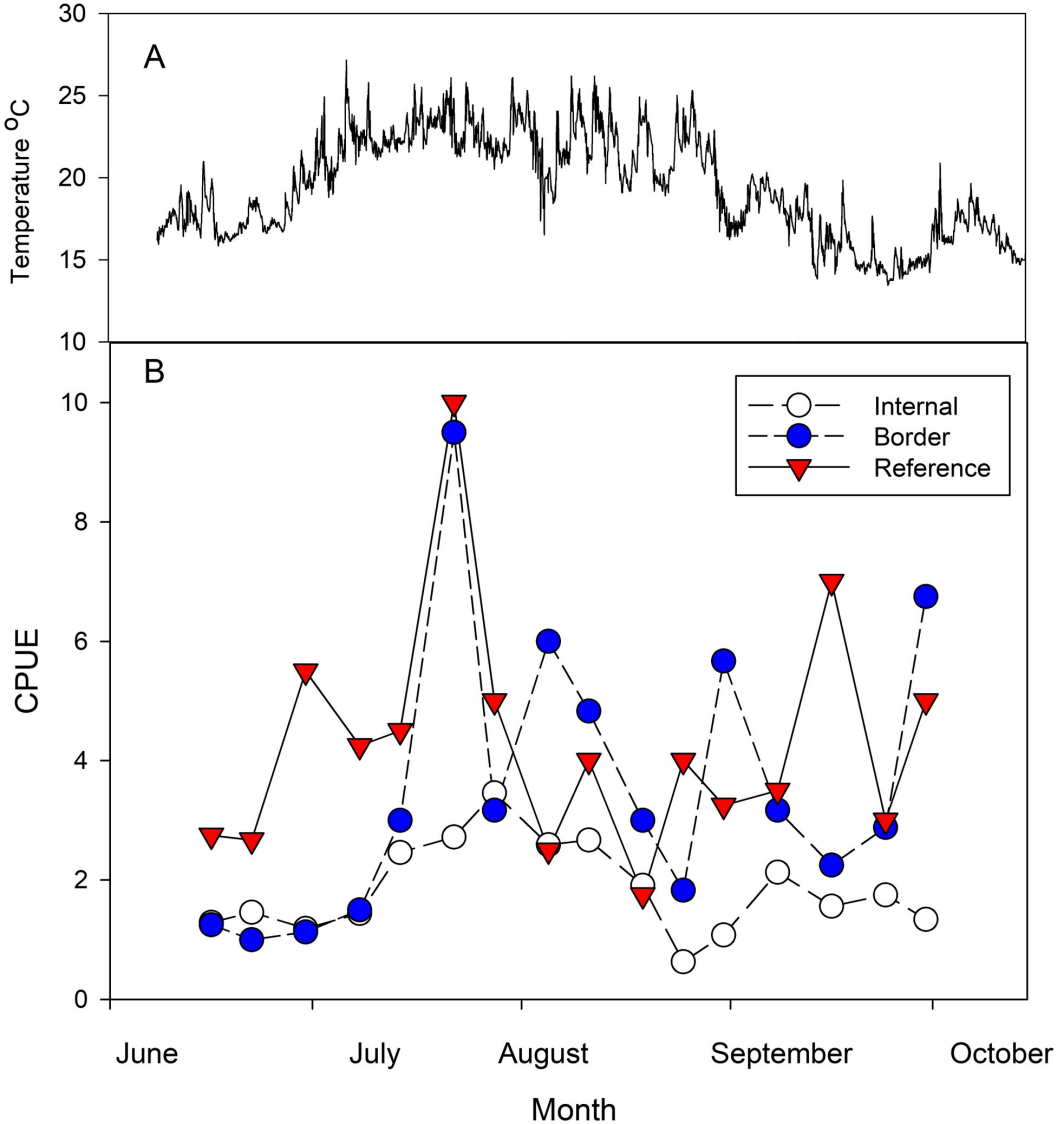
(A) Water temperature (°C) is plotted versus time. (B) The average catch per unit effort (CPUE) is plotted versus time. Each data point is connected by a straight line for illustrative purposes. Colors indicate trap location (white-internal; blue-border; red-reference).

Round gobies comprised 95.7% of the 1,689 fish trapped, with the rest of the catch consisting of 60 black crappie (*Pomoxis nigromaculatus*), five invasive tube-nose gobies (*Proterorhinus semilunaris*), four bass (*Micropterus spp*), three muskies (*Esox masquinongy*], one logperch (*Percina caprodes*), and nine crayfish (Cambaridae family). Water temperature ranged from 14.3 to 23.6°C throughout the study, with peaks in July and August (Fig 2A). The round goby CPUE steadily increased in all traps from June to mid-July before becoming more variable throughout August and September (Fig 2B).

**Fig 3:**
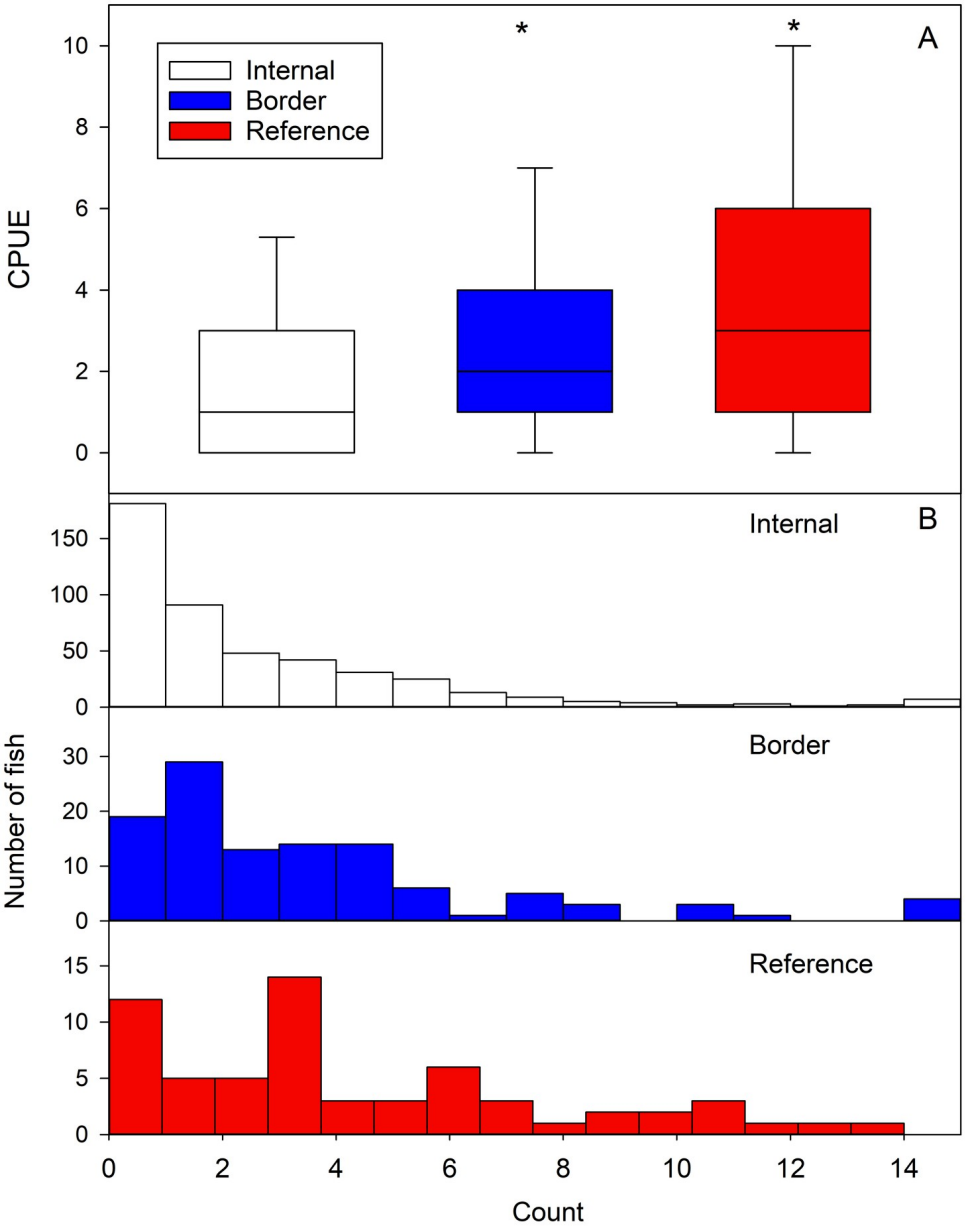
A) Box and whisker plots of the median CPUE (catch per unit effort) for internal (white), border (blue) and reference (red) traps with asterisks indicating significantly different medians (Kruskal-Wallis ANOVA, Holm-Sidak post hoc tests, P < 0.001). The median is shown as the horizontal line, the 25^th^ and 75^th^ percentiles are indicated at the end of each box, and the error bars represent the 10^th^ and 90^th^ percentiles. B) Histograms plotting the frequency of the number of round gobies found in each trap [internal (white), border (blue) and reference (red) traps]. Bin size is one except for captures ≥ 15 are reported in the last bin.

There was no significant difference in CPUE (Kruskal-Wallis ANOVA, H = 4.76, P = 0.19, DF = 3) between the four reference traps (R1 to R4), and data from these traps were pooled resulting in a median CPUE of 3.0 (Q1 = 1.0, Q3 = 6.3; range = 0 to 14). There was also no significant difference in CPUE between the two border traps (B1 and B10) (Mann Whitney Rank Sum Test P = 0.46, T = 3037.3, U = 1441.5), resulting in a combined median CPUE of 2.0 (Q1 = 1.0, Q3 = 4.0; range = 0 to 17). The internal traps (I2 through I9) had similar CPUEs (Kruskal-Wallis ANOVA P = 0.63, H = 1.73, DF = 7), allowing the data to be pooled and resulting in a median CPUE of 1.0 (Q1 = 0.0, Q3 = 3.0; range = 0 to 24). There was a significant difference in CPUE between the internal, border, and reference traps (Kruskal-Wallis ANOVA, H = 44.5, P < 0.001, DF = 2) (Fig 3A). A relatively higher percentage of internal traps (39.2%) were recovered empty compared to border (15.6%) or reference traps (19.4%), with over 60% of the internal traps containing either zero or one fish (Fig 3B).

**Fig 4:**
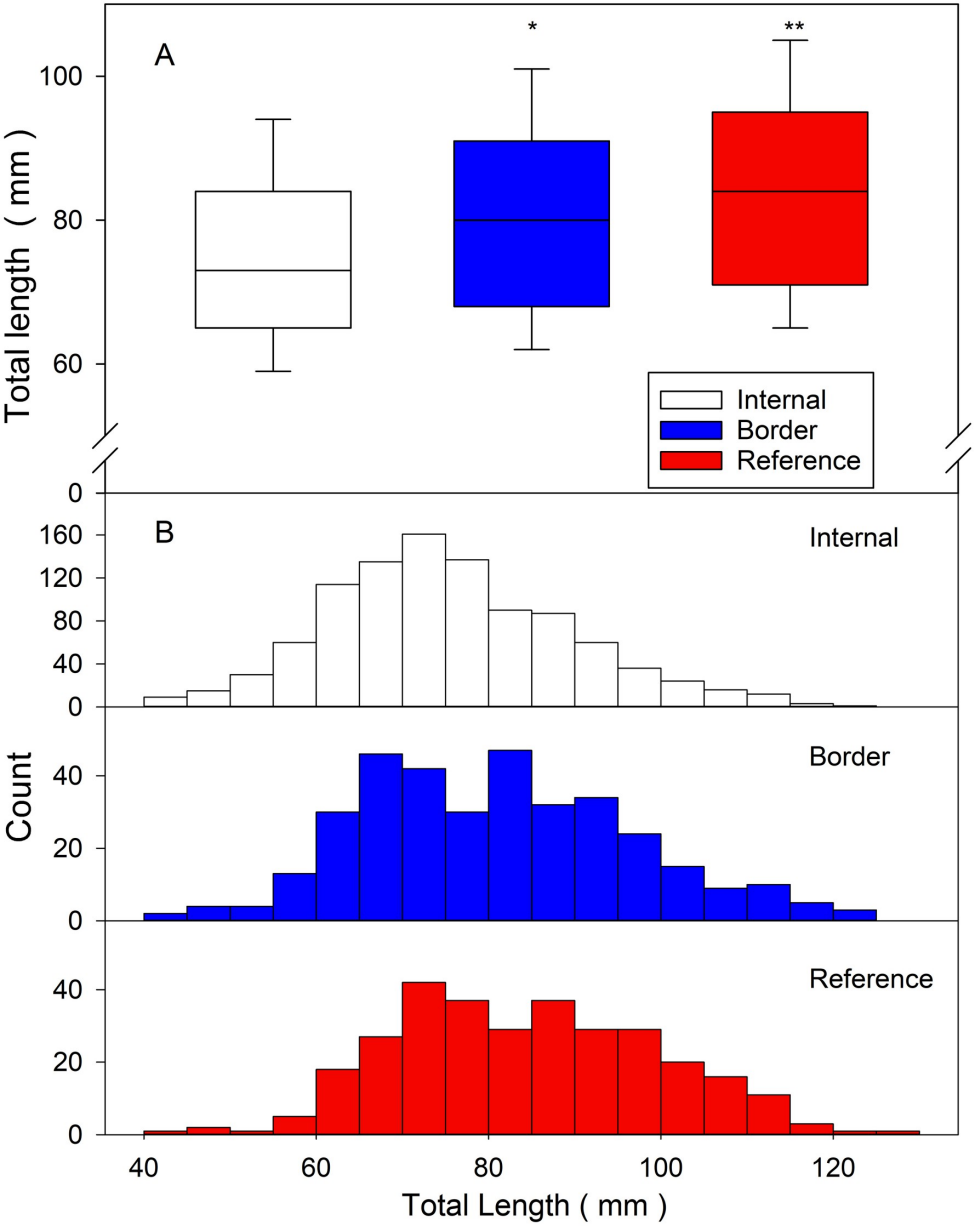
A) Box and whisker plots show the median total length for internal (white), border (blue) and reference (red) traps (Kruskal-Wallis ANOVA, Holm-Sidak post hoc tests *P < 0.05, **P < 0.001). The median is shown as the horizontal line, the 25^th^ and 75^th^ percentiles are indicated at the end of each box, and the error bars represent the 10^th^ and 90^th^ percentiles. B) Histograms plotting the size distribution of the total length (mm) of fish captured in each trap [internal (white), border (blue) and reference (red)]. Bin size is 5 mm.

The median total length of round gobies was significantly larger (Kruskal-Wallis ANOVA, H = 85,837, P < 0.001, DF = 2) in reference (median = 84 mm; Q1 = 71 mm, Q3 = 95 mm; range = 44 to 125 mm) and border traps (median = 80 mm; Q1 = 68 mm, Q3 = 91; range = 43 to 124 mm) than internal captures (median = 73 mm; Q1 = 65 mm, Q3 = 84 mm; range = 40 to 123 mm) (Fig 4A). The size histograms (Fig 4B) show the different total length distributions between the three groups.

**Fig 5:**
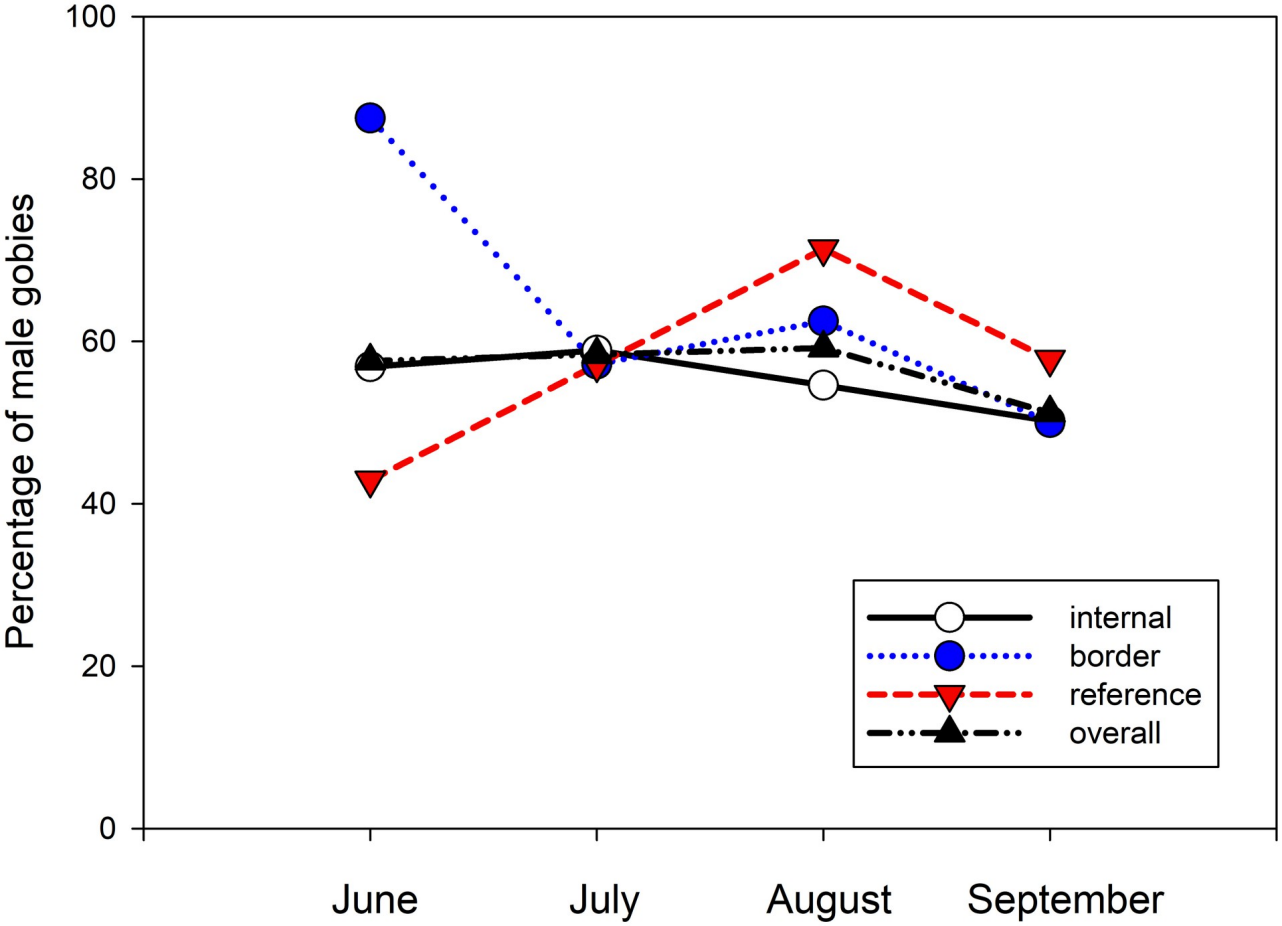
Percentage of male round gobies found in each trap [internal (white), border (blue), reference (red), and all traps (black)]. Data represent the percent of males captured in each category during the month indicated.

Throughout the study, 56.7% of the captured round gobies were male. Only the June reference traps contained more female than male gobies (42.8%) (Fig 5). However, by August the reference traps contained 70% males before declining to 57% by September. In comparison, the border traps contained 87.5% males in June that dropped to approximately 60% for July and August, ending at 50% for September. Internal traps ranged between 54.4% and 58.9% for the first three months of the study before declining to 50% in September.

From June 21st to September 29th, 2019, 167 of the round gobies captured in the reference traps were tagged (Table 1). Forty-two of these tagged fish were recaptured a total of 64 times. The majority of recaptured round gobies (82.8%) did not leave the area and were recaptured in the initial trap. Additionally, most recaptures (N = 55) were in reference traps (86.0%) with seven gobies found in the border traps and only two gobies captured in the internal traps (Table 1).

**Table 1:** Numbers indicate total tagged round gobies and recaptured at each site. R = Reference, B = Border and I = Internal.

Traps	Tagged/ Recapture	Tagged location			
		R1	R2	R3	R4
R1	58/23	22	0	0	1
R2	42/7	0	7	0	0
B1	NA	0	2	0	0
I2	NA	0	0	0	0
I3	NA	0	0	0	0
I4	NA	0	0	0	0
I5	NA	0	0	0	0
I6	NA	0	0	0	0
I7	NA	0	1	0	0
I8	NA	0	0	1	0
I9	NA	0	0	0	0
B10	NA	1	0	3	1
R3	25/8	0	0	8	0
R4	42/17	0	0	1	16

## Discussion

The CPUE was significantly lower in internal traps compared to border and reference traps. The internal traps also captured significantly smaller fish than the reference traps, indicating the traps were removing larger individuals. The percentage of males in the captured population slightly declined over the course of the study, yet male gobies still constituted most of the captures.

The study site was selected as it is representative of the rocky habitat throughout the Duluth-Superior Harbor, with high densities of round gobies and few native species [33]. Ice extends routinely from surface to substrate in the shallows which forces offshore seasonal migration of round gobies. The migration of round gobies into the area along the shoreline and from the deeper, offshore waters offered an opportunity to observe recolonization of the area. The reference traps were deployed less frequently than the interior traps to minimize the chance that the fish would acclimate to the food and alter movement pattern. The tagging results confirmed the round goby’s small home range [33, 38] with very few of the recaptured tagged fish displaying movement.

Based on previous observation of round goby longshore migration patterns [33], it was hypothesized that the border traps would intercept the fish tagged in the reference traps migrating parallel to the shore. It also was anticipated that the border traps would have a higher CPUE than the internal traps, which all were flanked by other traps that were actively removing round gobies while the border traps had only catch and release traps on one side. The border traps successfully captured most (7 of 9) of the migrators from the reference traps that swam towards the interior traps and had an intermediate CPUE compared to reference and internal traps.

The round goby population along rocky shorelines are characterized by large, sexually mature, and more aggressive fish that establish breeding territories and push smaller gobies into deeper water [35]. In the Duluth-Superior Harbor, the rocky inshore habitats quickly give way to sandy bottom and offshore dredged channels with less hard substrate. Round gobies trapped in the study area were larger than the average size of those trawled from offshore waters [33, 39]. It was therefore anticipated that regular removal of round gobies at the trap locations would encourage migration of smaller round gobies from deeper water into the area. The increased CPUE in border traps combined with the tagging data indicated that the border traps were intercepting shoreline migrators, suggesting that the internal traps were being populated by round gobies coming from deeper waters. The average total length of round gobies caught in the internal traps was significantly less than in the border and reference traps, which suggests recolonization primarily from the offshore population. The CPUE of the internal traps was significantly reduced compared to both the border and reference traps, strongly indicating that the round goby density was being reduced in the area. Additionally, 60% of the internal recoveries contained ≤ 1 fish, with these rates only observed in 42% and 27% of the deployments of the border and reference traps, respectively. Thus, the removal of fish from internal traps led to overall lower CPUEs indicating that the local density was declining.

The question remains whether the deployment periods were optimal. For example, longer deployment could yield a greater number of round gobies per trap and decrease labor needs, while daily trapping and rebaiting could further reduce the number of gobies in an area but require greater time investment. Another variable is that trapped fish may discourage other fish from entering the trap, however as several traps contained > 12 fish, this did not appear to be an issue, however the complete consumption of bait in high yield traps may have limited total catches.. Doubling the trap density in the experimental area would probably increase yields; however, trap deployment, trap reset, and data collection averaged approximately 8 minutes per trap [2 hours for 14 traps] with a two-person crew, and labor costs would need to be considered before adjusting removal frequency. While this investment may be manageable for a small area during a critical time window, it may not be cost effective on a larger scale.

A previous catch and release trapping study in this area showed a consistent 2 to 1 male to female ratio throughout the ice-free months from 2009–2010 [38]. As the males are responsible for aerating and defending the nest after spawning, male captures would leave the unguarded nest open to conspecific egg predation. Although the reference traps mirrored these trends, over the last three months of the current study, the border and internal trap’s capture of males decreased, indicating that either more males were being preferentially targeted or that the fish migrating into the area were predominantly female. The study was limited to a single breeding season due to the subsequent COVID-19 pandemic and we were unable to determine the effects on the subsequent year; however, the removal of sexually mature fish could have decreased the number of offspring the following year. Therefore, while intensive trapping did not remove all the round gobies, the impacts of round goby removal in an area may extend into the future and should be examined in subsequent years.

Removing round gobies with baited minnow traps proved to be a successful method for reducing the round goby density in a targeted area despite a lack of barriers to prevent round gobies from recolonizing the area. Thus, intensive targeted trapping can at least temporarily reduce round goby numbers. However, greater success could potentially be achieved if physical barriers such as nets were deployed to prevent the offshore population from migrating into the area. Previous studies have shown that adult round gobies move offshore during the winter months but migrate back to the same location on shore [33, 38].

While the size of the Duluth-Superior Harbor precludes any attempt to eradicate round gobies via trapping, the upstream migration of round gobies threatens critical spawning grounds for native fish such as lake sturgeon in the St. Louis River. The sturgeons spawn below the Fond du Lac dam in a limited area that would be amenable to round goby removal. Additionally, river trapping could be more effective as the downstream current may slow the rate of recolonization. In this specific scenario, trapping may reduce round goby number and predation on the eggs of native species [4, 13–16].

Several considerations need to be addressed with any trapping study. The current and previous studies [33] showed that the traps were effective in collecting large numbers of round gobies in a rocky area. However, the trap opening could exclude large fish and the mesh size may allow smaller fish to escape. The trapped fish ranged up to 130 mm TL, which was slightly below the maximum size captured during a previous trawling study (140 mm TL) [39]. Fish <4 cm were observed to escape the trap during collection however these are often sexually immature [39]. Two slightly different strategies were deployed in this study with the interior traps deployed every 48 hrs and their contents collected, while the reference traps were catch and release and deployed once per week for 48 hrs. In a previous catch and release study, using similar methodology in the same area, over 9,000 round gobies were trapped over two years with an average CPUE > 4 fish per trap [38]. There was concern individual gobies learned to associate the trap food as tagged fish were recaptured approximately four times. The lower frequency of recaptures in the current study (1.5 recaptures) indicated that weekly sampling may have mitigated this phenomenon.

## Conclusions

Our data suggests that removal by baited minnow traps reduces the relative abundance of round gobies in a shallow rocky area. As round gobies have been implicated in egg predation on native fish such as lake sturgeon [13], mottled sculpin [4], logperch [40], and smallmouth bass [16], these techniques could be used to reduce the invasive species near native fish spawning grounds. Additional investigations should examine optimizing temporal deployment of traps and subsequent recruitment following intensive trapping in an area.
